# Kinematic Strategies Underlying Improvement in the Acquisition of a Sequential Finger Task with Self-Generated vs. Cued Repetition Training

**DOI:** 10.1371/journal.pone.0052063

**Published:** 2012-12-18

**Authors:** Jason Friedman, Maria Korman

**Affiliations:** 1 Department of Cognitive Science, Macquarie University, Sydney, Australia; 2 ARC Centre of Excellence in Cognition and its Disorders, Macquarie University, Sydney, Australia; 3 Department of Occupational Therapy, Faculty of Social Welfare & Health Sciences, University of Haifa, Haifa, Israel; The University of Western Ontario, Canada

## Abstract

Many motor skills, such as typing, consist of articulating simple movements into novel sequences that are executed faster and smoother with practice. Dynamics of re-organization of these movement sequences with multi-session training and its dependence on the amount of self-regulation of pace during training is not yet fully understood. In this study, participants practiced a sequence of key presses. Training sessions consisted of either externally (Cued) or self-initiated (Uncued) training. Long-term improvements in performance speed were mainly due to reducing gaps between finger movements in both groups, but Uncued training induced higher gains. The underlying kinematic strategies producing these changes and the representation of the trained sequence differed significantly across subjects, although net gains in speed were similar. The differences in long-term memory due to the type of training and the variation in strategies between subjects, suggest that the different neural mechanisms may subserve the improvements observed in overall performance.

## Introduction

Many motor skills, such as typing or piano playing, evolve with the ability to articulate finger movements into novel sequences that are executed faster and smoother with practice. Considerable evidence indicates that a sequence of movements is organized as a unit [1]–[3] or a set of subunits: after a certain amount of practice has been afforded in the performance of a given movement sequence, component movements can be concatenated and reorganized into subsequences or ‘chunked’[1], [2], [4]–[8]. In this sense, serial keystrokes are analogous to speech movements and exhibit coarticulation - movements are planned and executed in parallel. The timing of these learned actions tends to be invariant, revealing the importance of temporal structure in motor skills, even when not essential to the meaning [9]–[11].

Skill learning and specifically the generation of long-term skill (procedural) memory are subserved by biological processes that must be triggered and allowed to proceed to a successful completion by the structure of the learning-training experience; during training as well as subsequent rest periods, activation in different brain regions changes dynamically [5], [12]. The performance of a given task is thought to reflect qualitatively different task solution routines in different phases of experience. Changes in procedural knowledge result in differences in the ability to transfer gains across stimulus, context and task parameters [13]. The process that occurs during the offline, between session periods, is referred to as consolidation, and is typically revealed either by increased resistance to interference (e.g., [13], [14]), and/or by subsequent improvement in performance [15].

While the performance of timing in procedural tasks has been explored extensively [16], [17], offline learning of temporal structure has been largely unexplored. Several recent studies however demonstrated that timing not only improved with practice but also revealed an offline benefit that was absent if the learner was sleep deprived (for a tracking task [18]; key-tapping and perceptual tasks [19]). A recent functional imaging study [20] has provided valuable insights into the brain structures mediating motor sequence learning, highlighting that general behavioural improvement in the early motor sequence learning of a key-tapping task is subserved by two distinct kinematic processes, related to velocity and transitions (between-movement gaps), whose behavioural expressions are supported by partially overlapping and segregated brain networks.

Several key factors are currently suggested to be critical for shaping the representation of coarticulated movement sequences in memory. The first is the number of elements in the sequence, defining the capacity to retain it as a chunk in working memory. Coding of longer keying sequences involves motor chunks for the individual sequence segments and information on how those motor chunks are to be concatenated [21], [22]. The second factor is the need to establish solid explicit or implicit knowledge of the acquired sequence [23], [24], that underlies an anticipatory effect of co-articulation and the susceptibility to sleep-dependent consolidation [15]. The third factor refers to the nature of experience - consistently executed specific sequence of movements may lead to coarticulation. The importance of the structural specificity of the sequence was recently highlighted in the report of Rosanov et al. [8], which showed that the gains attained in the performance of a well-trained sequence of finger movements relies on the order of the movements being exactly as practiced. In contrast, online programming of a new sequence from a set of highly trained movement segments, as in finger spelling [2], [25] or when each movement element of the sequence is initiated from a general start position, such as in touch typing [26] does not result in anticipatory modulations. Fourth, probably the most important factor affecting the representation of a movement sequence is the amount and structure of training. Some aspects, such as the significance of multi-session training are well established. Several studies suggest that coarticulation can occur in later stages of multi-session training. Traditionally, motor learning research has examined the ways that experimenters can manipulate practice to create a more effective learning experience, e.g., providing augmented feedback, sleep or massed practice.

Self-regulation during training was only recently recognized as an important variable in various types of motor learning experiments and aspects of the corresponding practice structures. These were tested in learner control over the frequency of augmented feedback presentation for both knowledge of performance [27] and knowledge of results [28]; frequency of model presentation for the learning of a badminton serve [29] as well as a basketball jump shot [30]; the task ordering of multiple tasks [31] and the online regulation of feedback during a continuous perceptual-motor task [32]. The results have generally shown that self-regulation is beneficial. To the best of our knowledge, the role of self-control of repetition rate during practice was not explored in the context of sequence learning.

Thus, it is still largely unknown how the interactions among finger movements that produce coarticulated anticipatory motion and coupling evolve and are further refined depending on the amount of self-control during training. Here, we explored the nature of the between-session improvements in a finger tapping sequence task in terms of movement kinematics and timing. Specifically, we tested how the type of training, self-generated repetition vs. cued repetition of a given amount of sequences, affected the temporal organization, overall effectiveness and the time-course across three sessions. In addition to investigating the temporal determinants of sequence learning, we were also interested in the representational status of the trained sequence. To achieve this, on the third experimental day we tested the ability of participants to generalize their experience under different transfer task conditions.

## Materials and Methods

### Ethics Statement

The study was approved by the Macquarie University Human Research Ethics committee. All of the participants gave written informed consent prior to commencement of the experiment, following the procedure approved by the aforementioned committee.

### Participants

A total of 15 young healthy subjects aged between 19 and 33 years divided into two groups of subjects (Cued group: 8 subjects, 5 female, average age 24±4 years. Uncued group: 7 subjects, 6 female, average age 25±5 years) participated in this study. Participants were recruited from an online subject pool. All subjects were strongly right-handed as assessed by the Edinburgh Handedness Inventory [33], reported no medication intake, no sleep complaint, and no psychiatric or neurological illness. Musicians and professional typists were excluded to avoid subjects with expertise on motor sequence task, although all subjects use a computer keyboard for everyday activities.

### Procedure

**Figure 1:**
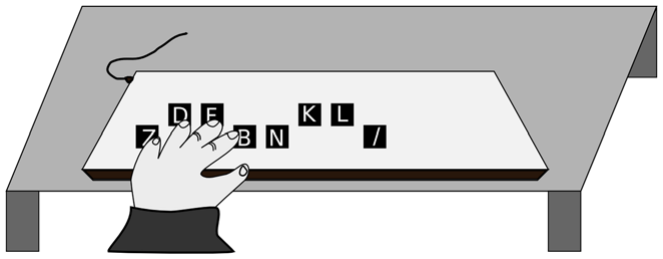
Experimental setup. The sequences were performed on a keyboard where the keys not used were removed. When using the left hand, 1 = index finger (B key), 2 = middle finger (F key), 3 = ring finger (D key), 4 = little finger (Z key). Optotrak infrared LEDs were attached using double sided tape to the fingernails of the four fingers.

A computerized version of the sequential finger-tapping task initially developed by Karni et al. [4] was used in the present study. Four keys, located in ergonomic positions on a standard computer keyboard were used (with keys-to-number assignment: where 1 = index finger (B key), 2 = middle finger (F key), 3 = ring finger (D key), 4 = little finger (Z key)). Similar to the protocol employed by Doyon et al. [34], the task consisted to repeat, as quickly and accurately as possible, a sequence of five finger movements using the left, non-dominant hand for a period of 30 s. Participants were given a number sequence to learn (4–1–3–2–4), which they repeated (see Figure 1). Keys not required for the task were removed from the keyboard. The experiment included three meetings of 40 minutes each, and was performed at the same time (in the middle of the day) over three consecutive days.

On the first day participants were familiarized with the sequence. When four correct sequences were executed, indicating that the subjects knew the sequence explicitly, the first session began. The session consisted of two pre-training tests, 10 blocks of training, and two post-training tests. Only two trials were used to assess the performance during the tests, minimizing the possibility that learning was induced from merely engaging additional practice (i.e., the test trials) and was not a result of type of training (cued or self-paced), leading to an offline gain. At the beginning of each block during the testing sessions, subjects were instructed to continuously tap the sequence as quickly and accurately as possible, immediately after hearing a “start” auditory signal until given a “stop” auditory signal thirty seconds later. Participants were instructed to look straight ahead, not at their fingers, and also not to practice the sequence outside of the experiment. During the test and training sessions, the screen stayed black and no feedback was provided. Participants were instructed that occasional errors should not be corrected, and were required to continue with the task without pausing.

There were two groups of subjects, each with different training protocols. For the Cued training group, training consisted of 10 training blocks, where each training block included 16 beeps, each 2.5 s apart. After each beep participants repeated the sequence once. For the Uncued second group, training also included 10 training blocks, but this time each block consisted of 16 self-timed repetitions of the sequence after a single beep, performed in a continuous manner, without a break between sequences. For both groups, the emphasis during training was placed on accuracy rather than speed. The interval between each training block was kept constant (30 seconds).

The second session on the next day was identical to the first, except that the initial familiarization with the sequence was not performed. On the third day, participants did not perform any training. They were tested in four different conditions. The first was the original trained sequence. Then three transfer conditions were tested: performance of the trained sequence with the untrained hand and of a reversed sequence of identical component movements with both the trained and untrained hand. The order of conditions was constant: The second was the reversed sequence (i.e., 4–2–3–1–4). The third was the original sequence on the (untrained) right hand. The fourth was the reversed sequence on the right hand. For the right hand, the index finger (1) used the N key, the middle finger (2) used the K key, the ring finger (3) used the L key and the little finger (4) used the ‘/’ key. Each condition was tested in four blocks of 30 seconds.

The number of correctly and erroneously typed sequences per 30 s test and transfer blocks was calculated.

### Data Recording

The experiments were run using the Psychophysics toolbox [35] and custom Matlab (The Mathworks, Inc) code. Key press information including timing was recorded, as well as the movements of the four fingers, using an Optotrak Certus motion capture device (Northern Digital, Inc.). An infrared LED was placed on the fingernail (using double sided tape) of each of the four fingers, and the 3D position of the fingers was recorded at 200 Hz.

### Data Analysis

The fingertip trajectory data was filtered using a 4^th^ order low-pass Butterworth filter with a cut-off of 20 Hz. The trajectory data was segmented into individual movements for each key press, using the timing from the key press data. The start of the movement was defined as the first time the tangential velocity was greater than 5% of peak velocity, and the end of the movement as the first time it went below 5% of peak velocity (after the time of peak velocity).

**Figure 2:**
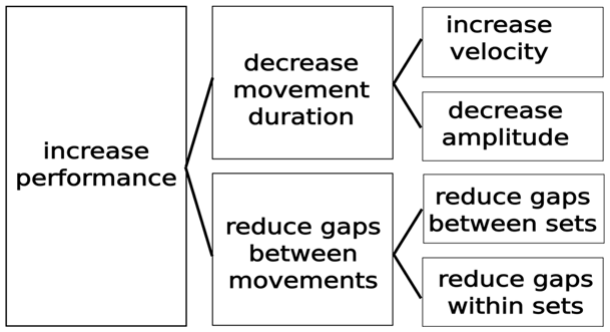
Decomposition of the improvement in performance (as measured in number of sequences performed). Errors are not considered in this diagram.

In order to normalise the data across subjects, we calculated the improvement, relative to the performance during the first test session. The improvement in performance can be achieved in two ways (in terms of kinematics, and assuming no change in the number of errors): (1) Decrease the duration of each movement; (2) Make the movements start closer to each other (coarticulate). These forms of improvement can be further broken down. The duration of each movement can be decreased by either decreasing the magnitude of the movement (while maintaining the same peak velocity), or by maintaining the same magnitude and increasing the peak velocity. A combination of these two is possible. The movements can be made to start closer to each other by reducing the pause between each movement. If there is no pause between the movements, one movement can start before the previous movement has finished. These methods of improvement are summarized in Figure 2.

The number of sequences *S_j_* that were completed in test *j* is equal to the amount of time available (*D* = 30 s) divided by the average time taken for each sequence in that test, *T_j_*. Then the relative improvement (compared to the first test session) *I_j_* is given by
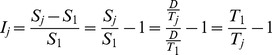



The time taken for each sequence can be divided into the time for the movements, *t_mj_* and the gaps between the movements *t_gj_*:
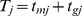



The improvement can then be decomposed into the part due to reduction in movement time, *I_mj_* and the reduction in gaps *I_gj_* (which sum to the total improvement):
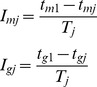



The improvement in the movement time can be further decomposed by considering a reduction of the amplitude of the movement or an increase in the peak velocity. We assume that these changes will result in a proportional change in the movement time, so the improvement due to an increase in peak velocity, *I_pj_* and the improvement due to a decrease in amplitude, *I_aj_* (given that the other quantity does not change) are given by
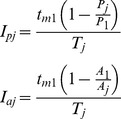
where *P_j_* is the peak velocity and *A_j_* the amplitude of the *j*th movement. We note that the sum of *I_pj_* and *I_aj_* will in general not be equal *I_mj_* because the effects of changes in peak velocity and amplitude are multiplicative.

### Statistical Analysis

Mean values are presented together with standard error. The extent of learning was statistically measured using repeated measures ANOVAs, with two groups as categorical values, and four training re-test time points (Post1, Pre2, Post2 and Post3) as within-subject factors. Repeated measures ANOVAs were performed on the overall performance (number of sequences performed), as well as the improvement due to change of movement duration and due to reduction of gaps. Post-hoc paired and unpaired t tests on the post-training and re-test scores were also calculated for each group. A p value <0.05 was considered significant. Two participants showed no learning or lapses in kinematic data acquisition and were thus excluded from the statistical analysis. Based on control experiments of the previous study, using a similar finger-opposition task [13], we assume that there are no baseline differences in the performance of the trained and the reversed sequences by both hands.

## Results

### Number of Sequences Completed (Non-normalized Data)

Two groups of subjects performed a computerized version of a five-element sequential finger-tapping task over three days, with different training regimes. During training, the Cued group participants were required to repeatedly perform a single sequence after hearing a beep, with a total of 160 repetitions during training. The Uncued group performed an equal number of sequences, but initiated each sequence themselves. Tests, performed before and after training sessions on two consecutive training days, included uncued performance of the sequence in a time window of 30 s for both groups. On the third day, the ability of participants to produce the trained sequence as well as to generalize their experience under different transfer task conditions was tested.

Both training protocols resulted in improved performance, measured by the number of correct sequences performed. The training on the first and second day resulted in average within-session increases of 4.8±2.0 and 2.9±1.9 sequences respectively for the Cued group, and 10.7±1.5 and 4.1±1.6 sequences respectively for the Uncued group (values indicated are standard error). One-sided paired t-tests showed that for the Cued group, only the first training was significant (t[7] = 2.34, p = 0.026), while for the Uncued group, both trainings were significant (t[6] = 7.22, p = 0.0002 and t[6] = 2.54, p = 0.022 respectively). On the group level, consolidation between testing days (i.e. between Post1 and Pre2, and between Post2 and Pre3) did not result in statistically significant increases in performance for either group (t-tests, p>0.05), suggesting there were no delayed off-line gains in performance between sessions, however, on an individual level some participants exhibited off-line learning: Cued group –2 participants out of 8; Uncued group –3 participants out of 7. Note, however, that for test measurement only two trials were averaged, this could impair our ability to reveal delayed gains in the speed of performance (such as in [36]) due to warm-up decrements in the first trial after daytime retention intervals (and consistent with [37]).

In terms of accuracy, no statistically significant difference in the absolute number of erroneous sequences was observed. Thus, no speed–accuracy trade-off was evident in either group. The correlation between performance rate and errors was negative for 5 of 8 subjects in the Cued group and 5 of 7 subjects in the Uncued group, further indicating that concurrent gains in speed and relative accuracy occurred. Moreover, the absolute number of errors remained constant at all three transfer tests. Further analysis focused only on the number of correct sequences performed, as the small number of errors biases the possibility to analyse learning of accuracy.

### Transfer

To probe the nature of the internal representations presumably subserving the large gains in performance triggered by multi-session training, the ability of participants to generalize their experience under different task conditions was measured during the third experimental day. There were significant practice-related gains for all transfer conditions relative to the baseline performance of the trained sequence for both the Cued (one-sided t-tests, RH: t[7] = 5.73, Rev: t[7] = 3.67, RH Rev: t[7] = 3.66, all p<0.01) and Uncued groups(RH: t[6] = 6.81, Rev: t[6] = 9.32, RH Rev: t[6] = 8.71, all p<0.01), consistent with our previous study in a finger-opposition task [13]. The effects of training were transferred almost completely to the right hand performance of the trained sequence both in terms of speed and accuracy (t-tests showed no significant difference between number of sequences performed between trained and RH for both groups), while a full transfer of gains to the non-trained sequence with the left hand was not found (one-sided paired t-tests showed significant differences for Rev [Cued: t(7) =  −5.22, p<0.001, Uncued: t(6) =  −3.82, p = 0.004] and for RH Rev for Uncued [t(6) = −2.88, p = 0.01] but not for Cued [t(7) =  −1.60, p = 0.08]). These results suggest that the gains after two training sessions were sequence-specific but not effector dependent.

### Normalized Data

For direct between-groups statistical comparisons, the normalized data was analysed. We defined the improvement in performance relative to the performance in the first test, e.g. an improvement of 1 means a 100% improvement, or equivalently, the subject performed double the number of sequences. There was a significant main effect for time points (F(3,39) = 6.64, p = 0.001) and a significant main effect of group (F(1,13) = 4.74, p = 0.049), but the interaction was not significant. This indicates that the participants in the Uncued condition showed significantly higher learning gains than those in the Cued group.

**Figure 3:**
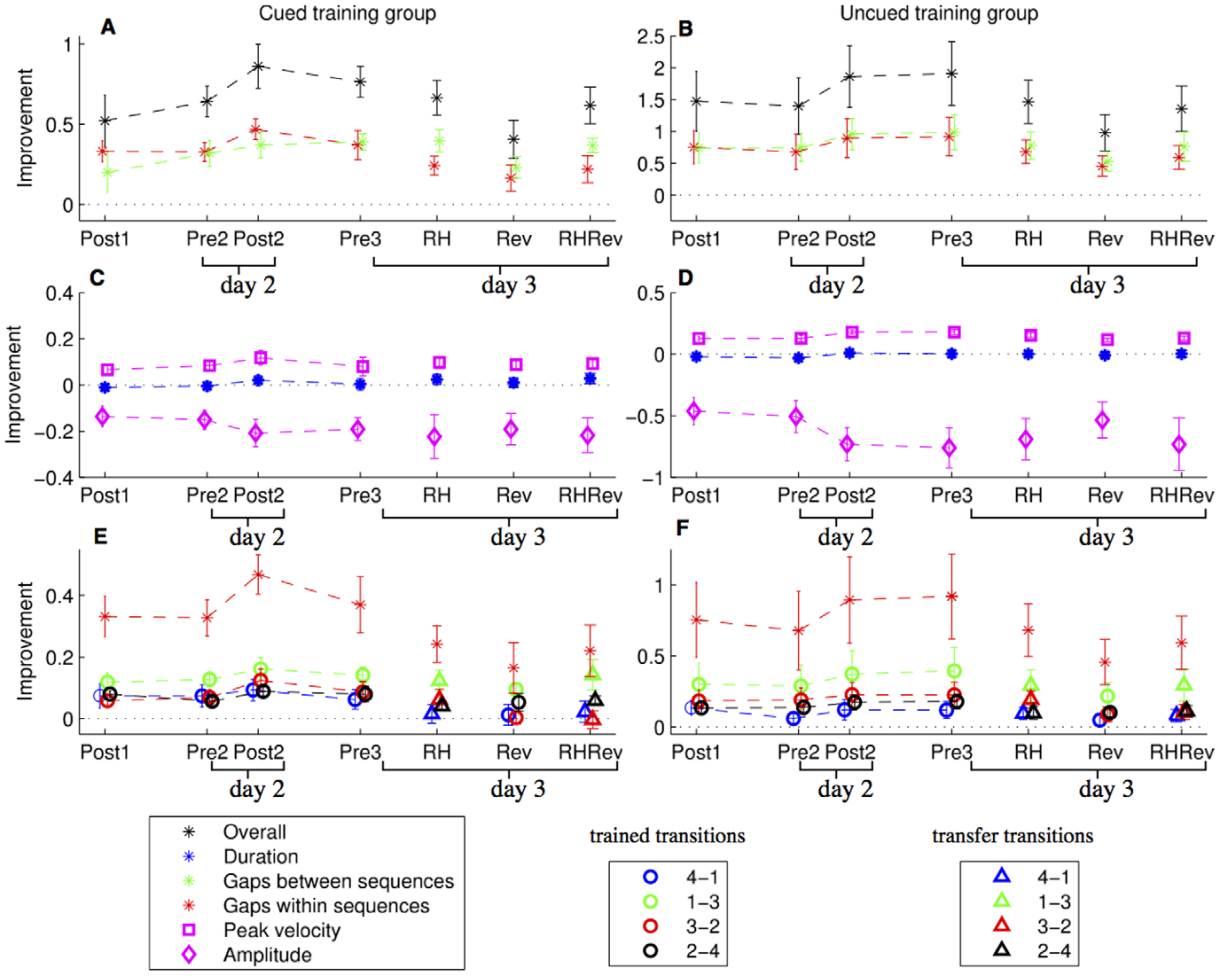
Mean improvement (relative to the first test session). Pre and Post refer to the tests before and after training. RH, Rev and RH Rev refer to the transfer conditions, right hand, reversed and right hand reversed respectively. The left column shows the Cued group, and the right column the Uncued group. The first row shows that the net improvement (in black) is primarily due to reducing the gaps within sequences (in red) and between sequences (in green). While the overall change in duration had a negligible effect, the decomposition of the duration in the second row into the contributions of peak velocity (square) and amplitude (diamond) shows that there were changes in the way the movements were made (faster movements, but with larger amplitudes). In the third row, the gaps within a movement are further decomposed into the gaps between the individual finger movements (circles), which sum to the total improvement due to reducing the gaps (in red stars). In both groups, the 1–3 transition was responsible for the greatest amount of improvement. The triangles indicate the gaps in the transfer conditions. We note that the scales are not equal across the groups.

To determine how subjects achieved this improvement, we decomposed the normalized improvement into its causes, as described in Figure 2. As is apparent from Figure 3(a–b), the magnitude of improvement in the Uncued group is approximately twice that of the Cued group (note the difference in scales between (a) and (b)). Both groups show approximately half of the improvement was due to reducing gaps within the sequence, and about half due to reducing gaps between the sequences (4–4 transition). Thus, for both training conditions, the main factor of improvement was the process of minimization of gaps between the finger movements, or coarticulation, whereas reduction in movement duration had a negligible effect.

### Movement Duration

The reduction in movement duration may be achieved by an increase in velocity or a decrease in amplitude of individual finger movements, or a combination of the two. Surprisingly, changes in duration minimally contributed to the improvements in performance speed in both groups. From the baseline pre-test, all finger movements were already extremely fast (mean 57±7 ms for the Cued group, and mean 56±8 ms for the Uncued group, standard errors reported) relative to the duration of a sequence (>1 s), presumably due to extensive experience of students with typing, leaving relatively little room for improvement. However, a repeated measures ANOVA on the duration showed a significant main effect for time (F(3,39) = 6.66, p = 0.001), suggesting that movement duration decreased with course of training. Moreover, t-tests between the time points showed a significant difference between Post2 (mean improvement of 0.015±0.018) and both Post1 (mean −0.015±0.019, p<0.001) and Pre2 (−0.017±0.018, p<0.001), suggesting that both within- and between-session, delayed gains in movement duration occurred in both groups, but not during the first training session.

We further decomposed these small changes in movement duration into the improvement due to the changes in peak velocity and amplitude (Figure 3(c–d)). The increases in peak velocity reduce the duration (one sided t-tests show the values are greater than zero for both groups; Cued: t[176] = 12.88, p<0.001. Uncued: t[160] = 24.25, p<0.001), whereas the increases in amplitude actually decrease performance (one sided t-tests show the values are less than zero for both groups; Cued: t[176] = −12.30, p<0.001. Uncued: t[160] = −16.41, p<0.001). A decomposition of movement duration into amplitudes and velocity improvements for each finger did not show any statistically significant differences (using a repeated measures ANOVA). However, for the amplitude, there were trends for differences between the fingers and the groups. In the Uncued group, the largest amplitude increases are observed for pressing the ‘4′ key at the start and end of the sequence, whereas for the cued group on average the increases in amplitude are different for the ‘4’ key pressed at the start and end of the sequence, suggesting very different representations for specific 4–4 transition are acquired through different types of training. These trends did not reach significance in ANOVA due to overall small size effect.

### Gaps between the Movements

We analysed how the between-finger time interval changed between specific transitions between finger movements during each production of a single trained sequence (4–1–3–2–4) within-sequence. For both groups, on average, the transitions 4–4 and 1–3 accounted for the largest improvement, as shown in Figure 3(e–f). Again, please note the scales of improvement in the two groups are different, with larger relative gains in the Uncued group. Remarkable differences were found between the two experimental groups in the transfer of gains. We quantified whether transfer took place by comparing the improvement between the Pre3 session and the transfer conditions, using one sided t-tests. When the t-tests are significant, the subjects performed significantly better in Pre3 than the transfer condition, so we assume that full transfer did not take place.

Although the contribution of transitions 1–3 and 4–4 for the improvement in trained condition was equal, the pattern of transfer of gains was unequal. For the Cued group, the gains for the 4–4 transition were transferred to RH (t[7] = −0.11, p = 0.54) and RH Rev (t[7] = 0.31, p = 0.38) but not to Rev (t[7] = 4.12, p = 0.002) conditions, whereas for the Uncued group there was no full transfer (Rev: t[6] = 3.58, p = 0.006; RH: t[6] = 2.42, p = 0.026; RH Rev:t[6] = 2.99, p = 0.012). The 1–3 transition showed full transfer for both groups for the RH (Cued: t[7] = 0.66, p = 0.27; Uncued: t[6] = 1.72, p = 0.07) and RH Rev (Cued: t[7] = −0.03, p = 0.51; Uncued: t[6] = 1.66, p = 0.07) conditions but not for the Rev condition (Cued: t[7] = 2.99, p = 0.01; Uncued: t[6] = 2.51, p = 0.02).

These findings suggest that by the third day the representation of the task was qualitatively and not only quantitatively (number of correct sequences) different between the Cued and Uncued groups, as highlighted by the different transfer of the 4–4 transition between the two groups.

**Figure 4:**
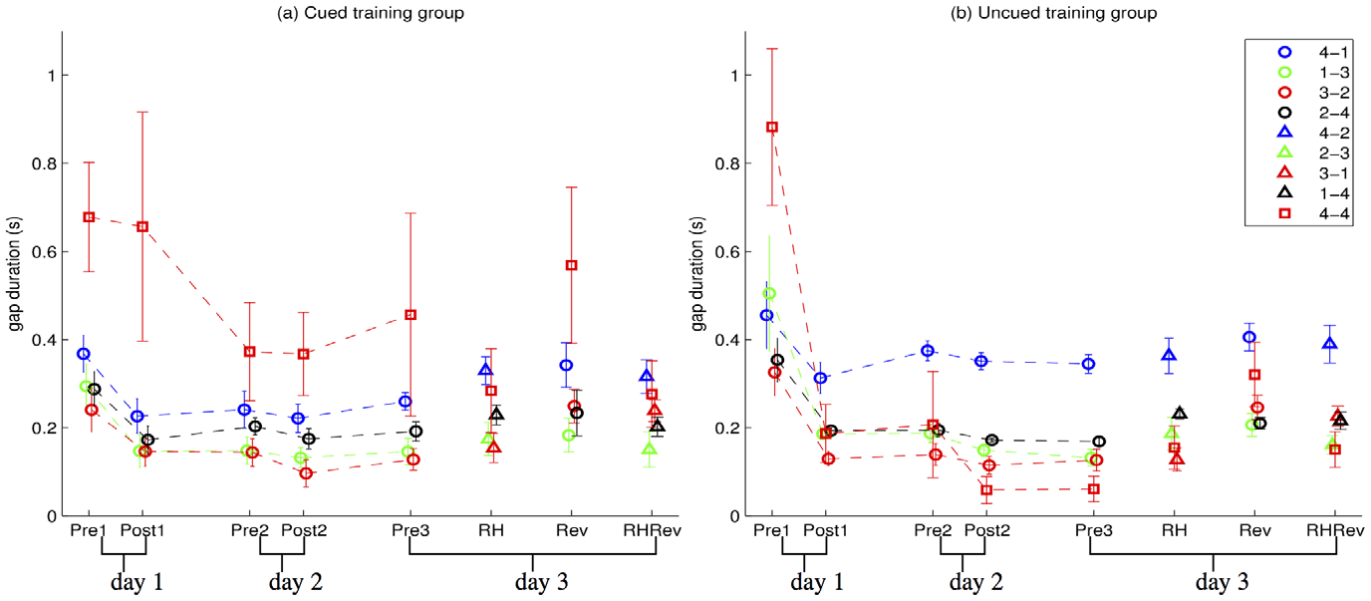
Mean gap duration for the two groups, for the between sequence gaps and within-sequence gaps, for all the transitions.

Figure 4 shows the mean gap durations for the two groups. At Pre1, the transition between finger 4 (sequence end) to finger 4 (next sequence start) was significantly slower than all other transitions in both groups. It was expected, as 4-to-4 movement pair can be produced only in a strictly serial manner. However, training of both types induced robust shortening in the time between these movements. A repeated measures ANOVA showed a main effect of time (F(4,52) = 5.541, p = 0.001), but no main effect of group or interaction. However, important differences in the time-course of gains were found: For the Cued group, most of the 4–4 transition improvement was between days 1 and 2, and the amount of improvement was similar to other transitions; 4–4 transition remained the slowest in the last test session. For the Uncued group, this transition became the fastest, with duration of only 61±31 ms at day 3 (compared to 883±192 ms in the first test). The improvement mostly occurred in the first training session, the difference in gaps was only significantly different between Pre1 and Post1. This remarkable reduction was the biggest effect among all tested finger movement pairs. Thus, qualitatively, the Cued group improved the 4–4 gap duration mainly between-session, showing transition-specific consolidation, whereas the Uncued group improved primarily within-session. This difference in 4–4 gap duration improvement can be interpreted as a direct temporal marker of the type of training. Cued training naturally emphasises the improvement within sequence “unit”, while Uncued training allows optimization of between units timing.

Training also significantly reduced the gaps between movement pairs 3–2, and 2–4 (paired t-tests at the 0.05 level between Pre1 and Pre3), reducing them on average from 280±28 ms to 127±17 ms (for 3–2) and from 319±32 ms to 181±12 ms (for 2–4), but not for the pair 4–1. For these within-sequence pairs most of the improvement happened within the first session and immediately post-training. This reduction in time between individual digit movement pairs was fully transferred to the complementary movement pairs (1–4 and 3–1) of the reversed transfer sequence by the fourth trial of performance. The slowest within-sequence transition in all training and transfer conditions was the initial transition (either 4–1 for the trained sequence or 4–2 for the reversed sequence).

### Individual Subject Performance

**Figure 5:**
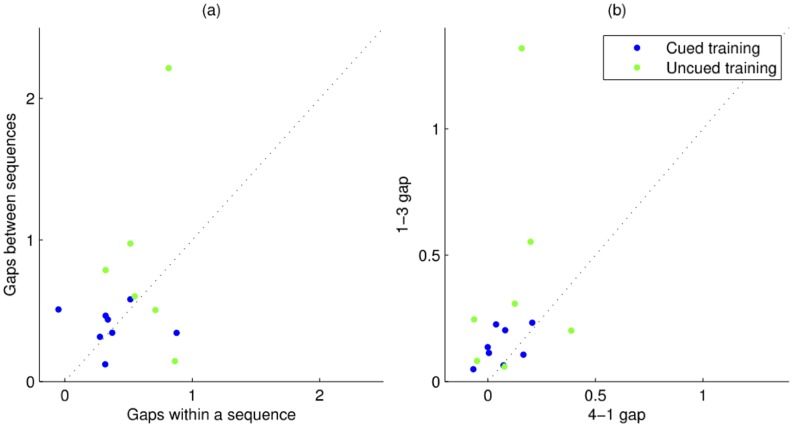
Summary of the individual differences in improvement across the subjects at Pre3. Each dot represents a subjects from the Cued group (in blue) or the Uncued group (in green). (a) The amount of improvement due to gaps between and within sequences is plotted. The dotted line represents an equal contribution of both sources. (b) The amount of improvement due to reduction of duration of the 1–3 gap and the 4–1 gap. Note that for some subjects, the improvement due to reduction in duration for the 4–1 gap is negative, i.e., this gap increased in duration.

While there were differences at the group level, there were also substantial differences between the ways that individual subjects improved their performance. In Figure 5, we have highlighted the major sources of improvement measured at day three test. Figure 5(a) shows scatter plot of the improvement due to gaps between sequences and gaps within a sequence, with each dot representing an individual subject. While group average improvements were approximately equal for gaps between and within sequences, on a subject-by-subject basis there was a large amount of variability in the strategies selected, demonstrated by the spread of the points. In Figure 5(b), we looked at the main sources of improvement within a sequence, reducing the 1–3 gap and the 4–1 gap. Again, a large amount of variability is observed, including negative values for some subjects (i.e., their 4–1 gap actually became longer).

**Figure 6:**
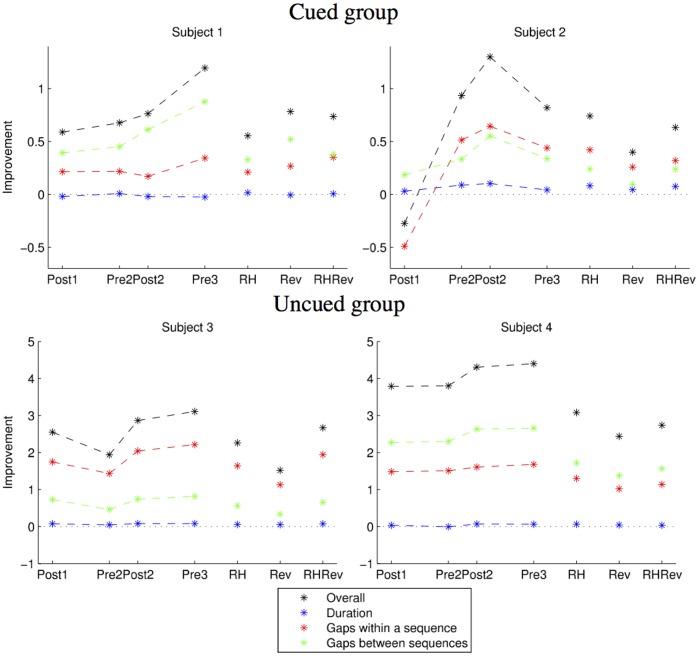
Improvment data from four subjects. Note the different scales between the subjects from the Cued group and the Uncued group. Each subject achieves the improvement in performance in a different way.

The individual pattern of relative contribution of the improvement in movement times and improvement due to reduced gaps between the finger movements to the performance of four individuals is further highlighted in Figure 6. During the course of training each subject adopted an individual combination of strategies, which were different not only with respect to the gaps between sequence movements but also with respect to the dynamics of their change within and between sessions. For example, in subject 1 there is a within session improvement (Pre2 to Post2) in the between sequence gaps concurrently with deterioration in within sequence gaps, while other subjects did not show such a trade-off. Importantly, by the third day of training, subjects did not converge to a general and presumably optimal solution for how to produce the sequence in either training condition. Instead, in most cases, optimization of the early set up representation was found. This suggests that early setting of the motor routine during the first session is of critical importance to the long-term outcomes of the multi-session training.

## Discussion

Successful performance of a sequential motor skill entails the correct execution of both the serial order and timing of the individual movements. In the case of typing, the order is clearly defined, whereas the “correct” or “optimal” temporal organization is a rather ambiguous question. The present study of skilled sequential key tapping indicates that people acquire the capacity to control sequence elements through collective treatment, e.g., activation in parallel. Such collective treatment may be a necessary condition for improvement in a task where each individual movement comprising the sequence is already executed very fast due to previous extensive experience with similar tasks (everyday typing). Our approach, combining behavioural measurements and an analysis of movements and their temporal structure, in the context of long-term training, allowed us to address the question of what are the substrates that underlie modification of a novel motor sequence representation.

Altogether, our results indicate that the knowledge gained from a training experience undergoes a number of important qualitative and quantitative changes that are dependent on the amount of self-regulation during the training sessions. Uncued group participants showed significantly higher relative learning gains in terms of the number of sequences completed compared to the Cued group, although both groups experienced the same amount and scheduling of practice. Experience exerted its effects on the speed of performance predominantly within the training session. Training beyond a single session resulted in sequence-specific but effector independent representation of a trained sequence in both groups. Thus, our results indicate an early setting up and time-dependent strengthening of a motor routine specific for the execution of the trained sequence of movements at an effector independent level. Why did the Uncued group improve more than the Cued group? One possible explanation is that the same neural mechanisms are involved in the two groups, but with differing temporal dynamics. To determine whether this is the case, we compared the ability of participants to generalize their experience under different task conditions. These data suggest that the two learning conditions resulted in qualitatively and not only quantitatively different task performance. Although we did not perform any direct neural measurements, it seems likely that these different patterns of learning reflect neural mechanisms that are partially segregated.

We broke down the overall improvement in performance into individual causes (see Figure 2). We found that almost all the improvement was due to reducing gaps between movements rather than decreasing the duration of the individual movements. This is probably due to the task – subjects were already extremely fast in making the movement, leaving little room for improvement, compared to the large amount of improvement possible in reducing the gaps. Despite this, we found that subjects increased their velocity and amplitude in a way that approximately cancelled out any net contribution to the improvement in speed, but may have led to increased tactile feedback (due to hitting the keys with a greater force). Studies of sensory feedback during tapping tasks [38], [39] and a piano playing sequence task [40] indicate that tactile information plays an important role in the control of timing.

We examined the differences in how pairs of movements are organized. Coarticulation appears to be a general feature of the assembly of finger key press movements, in that neural control is not biased to a strict serial ordering of the individual elements. Subjects exhibited anticipatory modifications of all specific movement pairs. However, the transitions 4–4 (the between-sequence transition) and 1–3 (the within-session transition) accounted for the largest improvement in both training groups. The gains in 4–4 gaps were remarkably different in their generalization ability between the groups: gains attained by day three on 4–4 transition in the Cued group were specific only for the movement sequence (transferred to the right hand, but not to the reversed sequence on both hands), whereas in the Uncued group the training gains were both sequence-specific and effector-dependent (no transfer). The 1–3 transition was not fully generalizable across hands in both groups. Thus, by the third day the representation of the task was qualitatively and not only quantitatively (speed of performance) different between Cued and Uncued groups. Altogether, our results indicate that an early setting-up of a motor routine, specific for the execution of the trained sequence of movements at an effector independent level, is still present after two sessions of training for the Cued type training (consistent with [13]), but not in Uncued training. Thus, the higher rate of learning gains in the Uncued condition may also reflect a faster shift in the representation of the acquired skill.

Although the 4- 4 pair of movements is the only strictly serially executed movement pair, it is a major factor influencing the total capability to achieve faster performance. The 4–4 movement pair is also the only one that remains unchanged during the production of the reversed sequence, and it was remarkable that in all transfer conditions in the Uncued group it was performed significantly slower than in the trained sequence. These observations further support the notion that the context of sequential movement production is an important parameter for effective behavioral expression of learning [8], [13]. Our results provide direct evidence for a unit-like representation of the trained sequence of movements in the nervous system. The fact that some transitions benefited more from training than others suggests that the temporal structure of the motor sequence underwent a learning-dependent within and between-session reorganization.

We did not succeed in triggering group significant delayed gains in performance speed as demonstrated for the finger-opposition version of the task [13], [14]. Several explanations may account for this. First, appearance of warm-up decrements after daytime retention intervals along with low number of trials averaged (two) could impair our ability to reveal delayed gains in speed of performance (consistent with [37]). Second, due to the similarity of the finger-tapping task to standard keyboard usage, executing a key press takes so little time that inter-key gaps are likely to reflect exclusively the underlying control mechanisms. As training induced improvement in the direction of novel coarticulation patterns, it is possible that alone they were insufficient to trigger delayed gains in performance speed on a group level (on an individual level some participants exhibited off-line learning).

Our study included fifteen subjects. Each exhibited an idiosyncratic pattern of temporal and kinematic parameters, and a unique pattern of modification with multi-session training, while having similar pattern of changes in behavioural parameters during the course of learning. We believe our findings reflect the normal range of movement strategies at early stages of sequential skill learning in young adults. In their Challenge Point Framework, Guadagnoli and Lee [41] introduced the concept of the learner as a central element around which practice should be structured. They suggested that motor skills not only have a nominal task difficulty (an amount that remains relatively constant across individuals and conditions) but also a functional task difficulty (a value that fluctuates according to the skill level of the individual performing the task as well as the conditions under which it is being performed). In this respect, we hypothesize that sequence learning task functional difficulty is reflected in the individual’s representation of temporal and ordinal features of a sequence, undergoing unique experience and time-based reorganization following multi-session practice. Importantly, we think that mixed patterns of temporal and kinematic parameters and their non-linear learning-dependent modifications explain the relatively high variability measured in terms of general behavioural correlates. Our conjecture is that further training to achieve highly skilled sequential performance may result in narrowing of the initially highly variable patterns of movements to some convergent effective strategy of movement execution for each individual subject, but not to some common solution on the group level. Further long-term multi-session study is needed to address this question.

Our results suggest that a learning-based reorganization of motor sequence representation is reflected in (changes of) specific temporal and kinematic measures of the executed movements, presumably along with changes in functional cortical motor representations during acquisition of new motor skills [5], [20], [42]. Different characteristics of the finger movements may independently change following training and exhibit specific time-course of learning-related modifications, as well as generalization abilities. Thus, although the execution of the finger-tapping task proceeds serially, we have presented evidence that movements are planned not in a strictly serial manner from the very beginning of learning and that this capability is acquired to an individual extent, depending on type of training and time.
